# Percutaneous tibial nerve stimulation (PTNS): an alternative treatment option for chronic therapy resistant anal fissure

**DOI:** 10.1007/s10151-019-01972-5

**Published:** 2019-04-10

**Authors:** Ursula Aho Fält, Martin Lindsten, Sara Strandberg, Mari Dahlberg, Salma Butt, Emelie Nilsson, Antoni Zawadzki, Louis Banka Johnson

**Affiliations:** 1 Department of Surgery, Pelvic Floor Centre, Skåne University Hospital-SUS, Lund University, 205 02 Malmö, Sweden; 2 Department of Surgery, Institution of Clinical Sciences Malmö, Skåne University Hospital-SUS, Lund University, 205 02 Malmö, Sweden

**Keywords:** Anal fissure, Percutaneous tibial nerve stimulation, Sacral nerve stimulation, Electrical stimulation, Wound healing

## Abstract

**Background:**

The aim of the present study was to evaluate percutaneous tibial nerve stimulation (PTNS) for treatment resistant chronic anal fissure.

**Methods:**

Consecutive patients with chronic anal fissure were treated with neuromodulation via the posterior tibial nerve between October 2013 and January 2014. Patients had PTNS for 30 min on 10 consecutive days. All patients had failed conventional medical treatment. The visual analogue scale (VAS) score, St. Marks score, Wexner’s constipation score, Brief Pain Inventory (BPI-SF), bleeding and mucosal healing were evaluated before treatment, at termination, after 3 months, and then yearly for 3 years.

**Results:**

Ten patients (4 males and 6 females; mean age 49.8 years) were identified but only 9 were evaluated as one patient’s fissure healed before PTNS was started. At 3-year follow-up, fissures had remained completely healed in 5 out of 9 patients. All patients stopped bleeding and were almost completely pain-free at 3 years (VAS *p* = 0.010) and pain relief improved from 50% at completion to 90% at 3 years. The patients’ Wexner constipation scores improved significantly (*p* = 0.007).

**Conclusions:**

In this small series, PTNS enhanced healing of chronic anal fissure and reduced pain and bleeding with an associated improvement in bowel function.

## Introduction

Chronic anal fissure (CAF) is a non-healing tear distal to the dentate line that has persisted for more than 6 weeks and characterised by pain and bleeding in and after defecation. The underlying pathophysiology is thought to be ischaemia exacerbated by spasm of the internal anal sphincter muscle (IAS) [1–3] and a chronic fissure being, in effect, an ischaemic ulcer in the anal canal [3–8]. More than half of CAFs heal with conservative medical treatment including bowel regulation and topical treatment with muscle relaxant ointments [1, 2], with less than < 10% healing without treatment aimed at reducing the anal spasm and hence improving blood flow [8, 9].

Lateral internal sphincterotomy (LIS) has in most countries been the gold standard treatment for CAF for many years. So far no non-invasive treatment has been able to show healing rates equivalent to LIS, the major drawback of the latter being the risk of permanent incontinence [1, 2, 10]. Neuromodulation has been suggested as an alternative, sphincter sparing treatment. Yakovlev et al. treated 5 patients with sacral nerve stimulation (SNS) for 3 weeks and achieved pain reduction and healing of fissure wounds in all participants up to 1 year after treatment [9, 11]. The effect of SNS on anorectal function occurs at a pelvic afferent or central level via S2–S4 nerves which contain fibres of the pudendus and afferent sensory nerves of the anal sphincter and pelvic floor. Based on this, Yilmaz et al. investigated the effect of a less invasive treatment with transcutaneous tibial nerve stimulation (TTNS) in 10 patients, who were treated for 10 days and achieved a 60% success rate which increased to 70% after further treatment sessions. The follow-up time was 2–9 months [12]. Posterior tibial nerve stimulation (PTNS) is another treatment option for stimulation of the tibial nerve, that like TTNS, is said to modulate the sacral plexus indirectly via the posterior tibial nerve, which contains sensory, motor and autonomic fibres from forth lumbar to third sacral roots (L4–S3). The mechanisms of its effect are yet to be fully elucidated, but extrapolation from SNS evidence would suggest both sensory and motor neuromodulatory effects [13]. Studies point to actions of SNS leading to non-specific activation of the enteric nervous system resulting in effects on both retrograde (antiperistaltic) and antegrade (propulsive) patterns or pressure waves [14, 15]. These actions lead to improvement in bowel movements [16]. Increased rectal mucosal blood flow has been also observed after PTNS and is seen as a marker of autonomic nervous function. SNS has also been shown to promote both an increase in rectal blood flow [17] and increase in intestinal mucosal healing rate after trauma [18]. Electrostimulation (ES) also causes increased activation of epithelial cells and keratinocytes, migration of fibroblasts and macrophages as well as an increased deposition of collagen with faster wound contraction [19–23].

No prior studies present data regarding the long-term results and recurrence rate after PTNS for CAF. Thus, we aimed to investigate the reproducibility of CAF healing with PTNS (primary endpoint) and the effects on pain, bleeding and bowel function with a follow-up of 3 years (secondary endpoints).

## Materials and methods

Patients presenting with CAF between October 2013 and January 2014 were treated with PTNS at our tertiary referral centre. The sample size was based on the number of units in a similar study [12], and the study was approved by the local ethics committee (Dnr 2016/998). Documented informed consent was obtained from the patients. Pre-treatment evaluation included detailed medical history and anorectal examination performed by one of two surgeon specialists. All patients presented with pain and/or bleeding, and the presence of a CAF was confirmed by clinical examination. Patients were included in the study if they were older than 18 years and had symptoms of CAF for at least 6 months. All patients had been on high-fibre diet and stool softeners and in addition failed conservative pharmacological treatment with topical application of anaesthetic creams, muscle relaxants (nitroglycerin or diltiazem) and/or botulinum toxin injection. Pregnant patients, as well as patients with a pacemaker device, low molecular heparin or warfarin treatment, neurological disease, inflammatory bowel disease, and/or radiation proctitis were excluded. The visual analogue score (VAS), St. Mark’s incontinence score, Wexner constipation score, Brief Pain Inventory-Short form (BPI-SF) were used for evaluation before and after treatment as well as assessment of bleeding and healing. VAS and BPI-SF were used to measure subjective pain characteristics and symptoms of patients at baseline (i.e. after topical treatment and stool softeners), after 2 weeks (end of treatment), 3 months and then once a year for 3 years. The St. Marks score and Wexner constipation score were used at baseline and 2 weeks, 3 months and 1 year after completion of treatment. Patients continued their high-fibre diet and stool softeners during the treatment. PTNS was performed by 2 experienced pelvic floor therapists using the Urgent PC Neuromodulation System^®^ (Uroplasty, Holland) on an outpatient basis. A stimulator provides electrical current with a fixed pulse frequency of 20 Hz, pulse width 200 µs and current setting between 0.5 and 9 mA (amplitude). A surface electrode was placed at the medial arch of the midfoot and a needle electrode was inserted through the skin posterior to the medial malleolus and advanced towards the posterior tibial nerve. Stimulation was gradually increased until a tingling sensation was perceived in the foot, or a motor flexor response was observed at the hallux. All patients received the treatment for 30 min 5 days a week, for 2 consecutive weeks. The fissure was evaluated before and after the last treatment session, as well as at 3 months and 1 year (though at the 3-month follow-up 1 patient declined clinical examination since he felt completely symptom free). These evaluations were performed by one of two surgical specialists. The fissure was characterised as not healed, partially healed when the healing process was visible but not totally complete, and healed when no sign of the fissure was visible. At 2 and 3 years, all patients were contacted by phone, and those who had symptoms which made it impossible to rule out recurrence or persistence of CAF attended for a clinical examination.

### Statistical analysis

The clinical outcome, i.e. none healed, partially or totally healed fissure, was analysed in relation to the VAS, mean St. Marks score and sum of the Wexner constipation scores. The Wilcoxon signed ranks test was used to analyse statistical significance. All analyses were performed with SPSS version 22 (IBM, Armonk, New York, USA). A *p* value < 0.05 was considered statistically significant.

## Results

Ten patients were included in the study (4 male and 6 female; mean age 49.8 years). However, 1 patient’s CAF had healed with conservative treatment before PTNS was started and was excluded from the analysis. Complete healing of CAF was observed in 9 out of 9 patients (100%) at 3-year follow-up; however, only 5 of these patients (55.5%) had healed after the 10 PTNS sessions alone. Of the remaining 4, 1 needed a total of 20 sessions and 3 patients presented with recurrent symptoms. One was treated with botulinum toxin injection 6 months after PTNS, 1 got a top-up PTNS followed by SNS within 6 months, and 1 patient was found to have an underlying fistula which was laid open 2 years after treatment with PTNS. All these patients were completely healed at 3 years. Only 1 patient had recurrent bleeding symptoms and investigation showed the source to be other than fissure disease. All 9 patients were almost completely pain-free at 3-year follow-up (the median VAS was 5 before treatment and 0 at 3 years, *p* = 0.010). Subjective pain relief improved after topical treatment and bowel regulation via stool softeners by 40% before PTNS treatment. After treatment, this increased to 50%. At 1 year, pain relief was significantly increased to 95% (*p* = 0.046) and 90% at 3 years (*p* = 0.046). The median St. Marks incontinence score was unchanged at 1 year. The median Wexner constipation score was significantly reduced (from 7.0 to 3.0, *p* = 0.007) at 1-year follow-up (Tables 1, 2, 3, 4).

**Table 1:** Fissure healing rate

	2 weeks (end of treatment)	3 months	1 year	2 years	3 years
No healing	2	–	–	1	–
Partially healed	5	3	2	1	–
Completely healed	2	5	7	7	9
Missing	–	1	–	–	–

The number of patients presenting with healed fissure on clinical examination and/or considered healed because of a complete absence of symptoms

**Table 2:** Presence of anal bleeding

	Before treatment	2 weeks (end of treatment)	3 months	1 year	2 years	3 years
Yes	6	2	4	0	0	1
No	1	3	3	9	9	8
Missing	2	4	2	–	–	–

**Table 3:** Pain and patient-experienced pain relief graded according to VAS and BPI-SF

	Before treatment	2 weeks (end of treatment)	3 months	1 year	3 years
VAS, mean/median (range)	4.3/5 (0–8)	3.7/3 (0–7) *p* = 0.465	3.3/3 (0–7) *p* = 0.102	0.4/0 (0–3) *p* *= 0.012*	1.2/0 (0–4) *p* *= 0.010*
Pain relief (BPI-SF) in %, median (range)	Baseline^a^ 40 (0–80)	50 (10–90) *p* = 0.098	40 (0–70) *p* = 0.593	95 (30–100) *p* *= 0.046*	90 (70–100) *p* = 0.046

^a^Baseline value after topical treatment and stool softeners

**Table 4:** Characteristics of bowel movements

	Before treatment	2 weeks (end of treatment)	3 months	1 year
Wexner constipation score, mean/median (range)	7.7/7 (4–14)	5.6/4 (2–16) *p* = 0.035	4.9/5 (0–14) *p* = 0.049	3.2/3 (0–7) *p* = 0.007

## Discussion

This pilot study with a long-term follow-up of 3 years shows promising healing rates and a low rate of recurrence for chronic anal fissure treated with PTNS.

At the end of 2 weeks of treatment, the CAF of 2 patients had healed and those of the remaining 5 were partially healed. At the 3-year follow-up, in 5 out of 9 patients, CAF had healed with PTNS as the sole treatment. When compared to the study performed by Yilmaz et al., the results are similar in terms of healing rate and reduction of pain. The follow-up in the study of Yilmaz et al. was for most patients limited to 2–4 months, with only 2 patients followed for longer (9 and 12 months).

Moya et al. treated 6 patients with CAF that had failed all other treatments including LIS, with PTNS. Fissure healing was observed initially in all 6 patients but in 3 cases fissure recurred after 6 months [24]. Ruiz-Tovar et al. randomised 80 patients with CAF to either glyceryl trinitrate ointment or PTNS for 8 weeks but without any prior attempt at conservative treatment. This resulted in a healing rate of 65% in the nitrate group and 87.5% for PTNS [25].

In this study, subjective pain relief increased from 40% at start of PTNS (following conservative treatment) to 50% at the end of treatment with PTNS and to 95% at 1 year. Other studies using ES have also reported a decrease in wound-related pain [19, 26]. The gate control theory by Melzack and Wall in 1965 [27] is the most widely accepted mechanism for pain relief in ES. This theory was revisited by Taniguchi et al. in 2015 and shown to be valid [28].

At the 3-month follow-up, 4 patients still had anal bleeding, emanating from partially healed but much less painful CAF. At 1- and 2-year follow-up, no patients reported any anal bleeding. At 3 years, 1 patient reported anal bleeding, but at examination with rigid sigmoidoscopy was found to be bleeding from haemorrhoids and not have a CAF.

Changes in bowel movements are most often associated with the onset of CAF, so before treatment all patients received stool softeners and or high-fibre intake to optimise stool consistency and frequency. The median value of the Wexner constipation score was significantly reduced from 7.5 to 3.0 (*p* = 0.007) at 1-year follow-up. This improvement in bowel function suggests that PTNS like SNS probably has an effect on colonic and anorectal motor function.

This study has limitations, primarily the small sample size and lack of a control group. Also it is difficult to draw more than association between treatment and relief of symptoms in such as small group using a technique with an unclear mechanism of action on CAF. We have not measured anal pressure or blood flow, which have been the two parameters most associated with CAF and healing. PTNS also requires specialist apparatus and skills, and so may not be generalisable as a treatment for CAF.

## Conclusions

PTNS may show early promise as a minimally invasive treatment for CAF, promoting long-term healing and a low rate of recurrence. The healing rate of approximately 56% 3 years following PTNS treatment alone indicates that PTNS may be used to reduce the number of patients needing surgery.

## Data Availability

The datasets analysed during the current study are available from the corresponding author on reasonable request.
